# Long non-coding RNA modifies chromatin: Epigenetic silencing by long non-coding RNAs

**DOI:** 10.1002/bies.201100084

**Published:** 2011-11

**Authors:** Alka Saxena, Piero Carninci

**Affiliations:** Omics Science Center, RIKEN Yokohama Institute1-7-22, Suehiro Cho, Tsurumi Ku, Yokohama, Kanagawa, 230-0045, Japan

**Keywords:** airn/Kcnq1ot1, enhancers, epigenetic modifications, non-coding RNA, Xist/HOTAIR, imprinting

## Abstract

Common themes are emerging in the molecular mechanisms of long non-coding RNA-mediated gene repression. Long non-coding RNAs (lncRNAs) participate in targeted gene silencing through chromatin remodelling, nuclear reorganisation, formation of a silencing domain and precise control over the entry of genes into silent compartments. The similarities suggest that these are fundamental processes of transcription regulation governed by lncRNAs. These findings have paved the way for analogous investigations on other lncRNAs and chromatin remodelling enzymes. Here we discuss these common mechanisms and provide our view on other molecules that warrant similar investigations. We also present our concepts on the possible mechanisms that may facilitate the exit of genes from the silencing domains and their potential therapeutic applications. Finally, we point to future areas of research and put forward our recommendations for improvements in resources and applications of existing technologies towards targeted outcomes in this active area of research.

## Introduction

Long non-coding RNAs (lncRNAs) are molecules often longer than 2 kb in length with a coding potential of less than 100 amino acids [1–3]. The number of lncRNAs exceeds that of protein coding genes [4–6] and their discovery has revolutionised the field of molecular biology.

Since their sequence provides no obvious clues regarding their function and the fact that they are poorly conserved across species, ncRNAs were viewed as non-functional and their presence and significance is still being debated [7–9]. However, as new studies identify the functions of individual ncRNAs, it is now apparent that many ncRNAs are the key regulators of transcriptional and translational output and therefore of cell fate and function [10–12]. While most mRNAs are exported to the cytoplasm for translation, many lncRNAs are now known to be retained in various sub-nuclear compartments [6, 13–15] suggesting that such RNAs may have a potential function in the compartment where they are localised.

Several nuclear lncRNAs have been studied in detail and investigations into the molecular functions of lncRNAs reveal more unexpected similarities in molecular functions than previously anticipated. Here, we will focus on some examples that provide important paradigms for gene regulation by lncRNAs through interaction with chromatin remodelling complexes. We believe that we have discovered only the tip of the iceberg of a hitherto unknown network of nuclear non-coding RNA/chromatin interaction.

Studies on four lncRNAs (*Kcnq1ot1, Airn, Xist* and *HOTAIR*) investigated individually by independent laboratories reveal that their function is to regulate transcription of multiple target genes through epigenetic modifications. These investigations have established fundamental principles of lncRNA function with broad implications. On the mouse X-chromosome, expression of lncRNA X-inactive specific transcript (*Xist*) from the designated inactive X-chromosome is essential for the silencing of the inactive X-chromosome [16–19]. On the Insulin like growth factor 2 receptor (Igf2r) imprinted cluster, located on mouse chromosome 17, the expression of paternal-specific non-coding transcript antisense *Igf2r* RNA non-coding (*Airn*, 108 kb), is required for the silencing of three genes on the paternal allele. These genes are spread over a large genomic region spanning 400 kb [20]. On mouse chromosome 7, the potassium voltage-gated channel subfamily Q member 1 (*Kcnq1*) imprinted cluster, spread over a 1 Mb genomic region in embryos, contains multiple genes and is silenced on the paternal allele by the un-spliced lncRNA *Kcnq1* overlapping transcript 1 (*Kcnq1ot1*, 91 kb) in cis [21, 22]. Some genes on the Homeobox D (HOXD) cluster, located over a 40 kb genomic region on human chromosome 2, are silenced by lncRNA HOTAIR, which originates from the HOXC cluster on chromosome 12 [23]. The elucidation of the molecular mechanisms of such long-range regulation reveals at least three common themes in the silencing process (Table 1).

**Table 1:** List of investigated long non-coding RNAs and their protein partners

lncRNA	Size (kb)	Spliced	Cover	Regulated genes	Escaped genes	Chromatin remodelling complex	Ref.
*Airn*	108	Yes	Yes	Multiple genes, in cis clusters	Yes, development specific escape	G9a	[20, 27]
*Kcnq1ot1*	94	No	Yes	Multiple genes in cis clusters	Yes, tissue specific escape	G9a	[22, 50]
						PRC2	
						PRC1	
*Xist*	17	Yes	Yes	Multiple genes in cis clusters on X chromosome	Yes, development specific escape	PRC2	[31, 67–69]
*HOTAIR*	2	Yes	Not known	Multiple genes in trans at HOXD locus, individual targets all over the genome	N/A	PRC2	[23, 29]
						LSD1	

## Silencing mediated by lncRNAs is imposed via recruitment of chromatin remodelling complexes

The involvement of RNAs in epigenetic silencing was proposed by various investigators [24, 25] based on the observation that while many enzymatic members of the chromatin remodelling complexes did not have DNA binding domains, they possessed RNA binding domains. Molecular investigations revealed the association between lncRNAs such as *Kcnq1ot1*, *Airn*, *Xist*, *HOTAIR* and chromatin remodelling complexes such as Polycomb repressive complexes 1 and 2, (PRC1 and PRC2) [22, 23, 26–32] which mediate mono-ubiquitinylation of Lysine 119 of Histone 2A (H2AK119ub)[33] and di- and tri-methylation of Histone 3 lysine 27 (H3K27me2 and H3K27me3)[34, 35], respectively; Lysine Specific Demethylase 1 (LSD1)/CoREST which demethylates mono- and di-methylated Histone 3 at Lysine 4 (H3K4) [36] and G9a histone methyl transferase which catalyses Histone 3 Lysine 9 di- and tri-methylation (H3K9me2 and H3K9me3) [37, 38].

**Figure 1:**
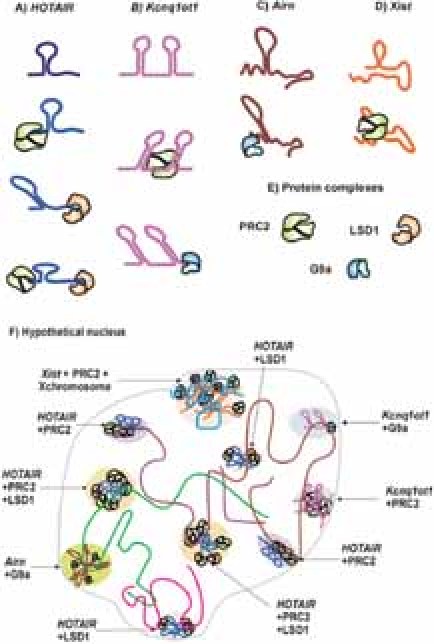
Complexity in lncRNA-chromatin modifying complex interactions. Chromatin remodelling complexes are capable of interacting with multiple lncRNAs. Similarly, lncRNAs may interact with more than one protein complex. **A** to **D:** Protein lncRNA interactions may result in conformational changes, which may help distinguish target specificity. **E:** Chromatin remodelling complexes **F:** Hypothetical nucleus depicting lncRNA–protein complexes and their silencing compartments. *Xist* domain is shown at the nuclear periphery with inactive X chromatin. *Airn* and *Kcnq1ot1* are shown to silence specific genes on their respective imprinted loci while *HOTAIR* is seen to target loci genome wide in concert with different protein complexes. Note that mouse *Hotair* does not participate in silencing the *HoxD* cluster and is not reported to interact with chromatin remodelling complexes [55]. Human HOTAIR is depicted in this schema.

At the *Kcnq1* imprinted locus, *Kcnq1ot1* lncRNA interacts with histone methyltransferase G9a and members of the PRC2 complex [22]. In addition, Terranova et al. reported close proximity between *Kcnq1ot1* lncRNA and members of PRC2 and PRC1 complex [28]. At the *Igf2r* imprinted locus, *Airn* also associates with G9a [27]. The imprinted genes in the *Igf2r* and *Kcnq1* clusters show repressive histone marks of K3K9me3 and H3K27me3 most likely induced by G9a and PRC2-remodelling complexes, respectively [22, 27]. It should be noted that these studies were performed in extra embryonic placental tissue in mouse and the mechanisms of imprinting in embryonic tissues may be different. On the X-chromosome, *Xist* lncRNA interacts with Ezh2 and Suz12 components of the PRC2 complex via a repeat A region (RepA) and the recruitment of PRC2 to the inactive X-chromosome induces the repressive epigenetic mark of H3K27me3 [31]. At the HOXD locus, *HOTAIR* also recruits PRC2 complex to induce silencing of specific genes [23]. It is noteworthy that at some of the loci mentioned above, the target genes fail to be silenced in the absence of the lncRNA [20, 23, 31, 39] thus implying that lncRNAs are essential for steering chromatin remodelling complexes to distinct target sites in order to induce silencing. Since these complexes interact with multiple lncRNAs, it appears that association with lncRNAs defines their target specificity. For example the repressive complex G9a, in concert with lncRNA *Airn*, targets the *Igf2r* imprinted locus, while in association with *Kcnq1ot1*, G9a represses genes in the *Kcnq1* locus [22, 27]. Similarly PRC2 in association with *HOTAIR*, targets the HOXD locus [23]; with *Kcnq1ot1* it targets the *Kcnq1* cluster [22], and while associated with *RepA/Xist*, it modifies histones on the X-chromosome [31]. Thus each protein complex is capable of being directed by multiple lncRNAs (Fig. 1 A–D). However, it is not clear if members of chromatin remodelling protein complexes have distinct domains for binding with specific ncRNAs or whether they bind in general to ncRNA molecules presenting certain secondary structures as seen in Xist [31, 40]. Indeed, it has recently been reported that short RNAs (50–200 nt), transcribed from repressed loci by stalled RNA polymerase II, interact with the PRC2 complex through their stem loop secondary structure and mediate gene repression through epigenetic modification [41]. It is not known whether the other ncRNAs such as Promoter associated short and long RNAs (PASRs, and PALRs) and promoter upstream transcripts (PROMPTs) generated around promoters as well as the vast numbers of small RNAs now known to be retained in the nucleus [6, 15, 42–44] possess distinct secondary structures and participate in local epigenetic regulation through interaction with chromatin remodelling complexes.

Interestingly, some lncRNAs also appear to interact with more than one chromatin-modifying complex. For example *HOTAIR* is known to interact with both PRC2 and LSD1/CoREST/REST complexes [29] and *Kcnq1ot1* interacts with G9a as well as the PRC2 complex [22, 28, 30]. Recent reports identify ANRIL (antisense non-coding RNA in the INK4 locus) as another candidate lncRNA which interacts with more than one chromatin remodelling complex to induce silencing in cis [45, 46]. ANRIL (3.8 kb) originates close to the INK4A gene on chromosome 9 in humans and interacts with the CBX7 component of the PRC1 complex to induce silencing of the INK4A and INK4B loci [46] and with SUZ12 component of the PRC2 complex to mediate epigenetic silencing of the p15^INK4B^ gene [45]. Thus the interaction of a single ncRNA with multiple chromatin modifying complexes to target specific genes may be a widespread phenomenon.

Indeed, in a high throughput RIP-Chip analysis, Khalil et al. found that 40% of long intergenic ncRNAs (lincRNAs) associated with the CoREST complex were also associated with the PRC2 complex, indicating that lincRNAs can have shared and independent targets [26]. Genome wide ChIP-Chip analysis of human promoters reveals 4,740 and 2,116 gene promoters occupied by PRC2 and LSD1, respectively, while 721 promoters are occupied by both complexes, suggesting shared and individual targets of each complex [29]. It is likely that repression at shared targets is mediated by ncRNAs capable of binding more than two complexes as seen with *HOTAIR* [29]. Thus it is now apparent that target specificity of lncRNAs can also be altered depending on the interacting chromatin modifying complex. These multiple interactions between chromatin complexes and lncRNAs may be sequence dependent as in the case of HOTAIR which has distinct domains for interaction with PRC2 and LSD1 [29]. However, the target distinction of the lncRNA-chromatin remodelling complexes is most likely mediated by conformational changes induced by these interactions (Fig. 1). It should be noted that while the ncRNAS mentioned above are certainly required for the initiation of silencing at their respective targets, it is not yet clear if like *Xist*, they are dispensable for the maintenance of silent epigenetic state at their target loci.

Additional mechanisms of ncRNA mediated silencing may exist in a gene or tissue specific manner. Certainly, *Airn* utilises gene specific silencing mechanisms even within the placenta, the *Slc22a3* gene is silenced through recruitment of G9a, however silencing of the neighbouring *Igf2r* gene does not require G9a since its imprinted status is not affected in G9a KO mice [27]. In mouse ES cells, the *Igf2r* gene is persistently expressed at low levels from the paternal imprinted allele despite DNA methylation at its promoter [47], indicating that *Airn* transcription itself may interfere with transcription initiation at *Igf2r* [48, 49]. At the *Kcnq1* locus, ubiquitously imprinted genes (genes imprinted in placenta, embryo and adult tissues) are silenced in the placenta and liver by recruitment of Dnmt1 by *Kcnq1ot1* [50]. The observation of imprinting and X inactivation phenomena, despite the poor conservation of ncRNAs such as *Airn* in opossum and dog [51, 52] and *Xist* in marsupials [53, 54] and the presence of a dysfunctional, poorly conserved *Hotair* in mouse [55], suggests the existence of compensatory layers of gene regulation in such species. It is likely that other modes of silencing may also emerge as common mechanisms for ncRNA-mediated silencing. Alternatively, the functional module of the lncRNAs in such species may be much shorter and dependent on secondary structure rather than length or primary sequence.

## The promoters of genes silenced by lncRNAs are covered with lncRNAs

A physical association between lncRNAs and the chromatin of their target loci is emerging as a common theme for very long ncRNAs that silence genes in clusters. *Xist* was first shown by RNA-FISH studies to coat the chromatin at the inactive X chromosome in a non-uniform manner, where euchromatic regions on the inactive X remained devoid of *Xist* coating in the initial stages of X-chromosome inactivation [56–58]. The physical association of *Xist* with chromatin was further confirmed by immunoprecipitation with antibodies against macroH2A1, a histone H2A variant enriched on the inactive X chromosome [59] (reviewed in [60]). On the *Kcnq1* locus, RNA-DNA-FISH studies reveal that *Kcnq1ot1* also associates with imprinted genes on the *Kcnq1* imprinted chromatin region [22, 61, 62] and at the *Igf2r* locus. Nagano et al. demonstrated through RNA-DNA-FISH a cloud of ‘*Airn*’ over the imprinted *Slc22a3* at E11.5 [27]. Such a ncRNA-cloud has not yet been reported for *HOTAIR*, which mediates silencing in trans and is only 2 kb long. Current data are insufficient to conclude whether this covering of targets is the exclusive property of long ncRNAs acting in cis over clustered targets.

The coating of lncRNA over targeted genomic loci is postulated to create a ‘silent nuclear compartment’ resulting in the recruitment of chromatin remodelling complexes to maintain silent chromatin marks and restrict access to the transcriptional machinery [63]. At the sub-nuclear level, such silent compartments created by *Xist* and *Kcnq1ot1* cover have been reported to localise to the perinucleolar region [22, 64, 65] suggesting that lncRNAs may induce changes in the spatial organisation of chromatin in the nucleus. It is notable that the lncRNAs involved in the formation of these silent domains are cis acting, particularly long (over 10 kb) and known to silence genes spread over large genomic loci on single chromosomes. It is not known if the many small RNAs retained in the nucleus participate in the formation of such clouds, since it is difficult to visualise small RNAs with current techniques such as FISH. Chow et al. recently reported the presence of small siRNAs arising from the young LINE1 elements in the *Huwe-1* gene, which facilitate its silencing by inclusion into the *Xist* domain [66]. Kanhere et al. reported sRNAs arising from PRC2 target genes that participate in the recruitment of PRC2 to their promoters [41]. It will be of interest to investigate if these short RNAs remain in the vicinity of their target loci and participate in the formation of a cover.

## Silenced genes enter while active genes remain outside the lncRNA silencing compartment

The location of genes under the cover of lncRNAs appears to be dynamic. Detailed analyses reveal that the genes that undergo X-inactivation gradually relocate deep inside the *Xist-*covered silent compartment as they are silenced [63]. Genes that escape X-inactivation remain outside this silent domain [63]. It was recently shown that inclusion of genes within the *Xist* silencing compartment is dependent on the density and proximity of young full length long interspersed nuclear elements (LINEs) to the genes [66]. However, the evidence from genes such as *Jarid1c (Kdm5c/Smcx)*, *Shroom2* and *Mid1*, which undergo tissue or development stage specific inactivation [67–69], suggests that in addition to the abundance and proximity of LINEs, other tissue/development-specific factors may also play a role in facilitating inclusion into the silencing compartment. *Jarid1c* is a LINE poor gene [66], which initially undergoes X-inactivation but is activated at later stages of development [67]. *Jarid1c* is expressed at equal levels in males and females in neonatal brains and adult liver [70] but escapes X-inactivation in adult female brains [70]. RNA-DNA-FISH studies reveal that *Jarid1c* remains at the inner edge of the Xist silent compartment in cells where it is inactive and it is located outside the *Xist* compartment in cells where it is active [63]. Another gene *Shroom2*, undergoes X-inactivation and displays PRC2-mediated H3K27me3 mark of repressive chromatin in a tissue specific manner; while *Mid1* gene shows H3K27me3 enrichment in female embryos but not in adult liver, indicating that it undergoes X-inactivation in embryonic tissues and later escapes X-inactivation [69]. Although RNA-DNA-FISH data for *Mid1* and *Shroom2* are not available, it is plausible that these genes are also located inside the Xist silencing compartment when they undergo X-inactivation. To escape inactivation *Mid1* and *Shroom2* would have to exit the silencing compartment, since the transcription machinery is located outside this compartment.

The exit of genes from lncRNA silencing domains is also seen at the imprinted loci. The *Slc22a3* gene is imprinted in embryos at E11.5 but shows biallelic expression at E15.5 [27]. Using RNA-DNA-FISH techniques, Nagano et al. demonstrated *Slc22a3* inside the silencing compartment under cover of *Airn* at E11.5 when it is imprinted and a reduction in *Slc22a3* loci covered by *Airn* at E15.5 after escape from imprinting [27]. The interaction between *Airn* and the *Slc22a3* promoter demonstrated by RNA TRAP experiments at E11.5 was reduced at E15.5 [27]. On the Kcnq1 cluster, genes are differentially regulated in placenta and embryos at E12.5 and studies of the lncRNA cover index over such genes suggests that only silenced genes remain inside the inactivation domain [61]. Interestingly, the imprinted loci are not particularly abundant in LINEs [71] suggesting that factors other than LINEs regulate the inclusion of genes in the silencing compartment at the imprinted loci. The fact that on the X-chromosome, as well as the imprinted loci, genes can escape from the silencing compartment into the transcriptionally active domains, despite the presence of the perpetrating lncRNA and repressive chromatin complexes in the vicinity, also suggests an additional layer of regulatory control that governs exit from the silencing compartment. Furthermore, tissue/development stage-specific silencing of X-linked and imprinted genes [67, 69, 70, 72] also argues against genomic features as key regulators of entry into silencing domains and suggests that at certain loci inclusion into lncRNA silencing compartment may be regulated by other factors responsive to development stage or tissue specific molecular signals. Intriguingly, the abundant expression and retrotransposition of LINE-1 in neuronal precursor cells is postulated to create gene disruption and diversity in the genome [73].

It will be of interest to investigate if such LINEs also play an active role in gene silencing by facilitating the influence of lncRNAs.

## Possible role for enhancers in escaping epigenetic regulation mediated by lncRNAs

It is now apparent that genes once silenced by inclusion into the silent domains of the lncRNAs are capable of reactivation in a tissue or development stage specific manner.

This reactivation most likely requires the genes to escape from the silent compartment. What regulates the exit of genes from the silencing domains created by lncRNAs? For such regulation to be effective, the controlling mechanism must remain outside the influence of the silencing compartment mediated by lncRNAs and be able to respond to developmental cues. Genomic regions called enhancers meet both requirements and are likely candidates for such regulation. Enhancers are DNA elements which provide binding sites for sequence specific transcription factors and induce transcription by facilitating the recruitment of RNA pol II to promoters (reviewed in [74]). FISH and 3C studies have shown that enhancers activate transcription in cis and in trans and that transcription activation by enhancers requires physical contact with the promoters via chromatin looping [75]. It is now known that distal elements bound by p300 with a chromatin signature of high H3K4me1 and H3K27ac and low H3K4me3 marks enhancers while core promoters are nucleosome free regions flanked by high H3K4me3 and bound by RNA polymerase II [76, 77]. About 25% of enhancers are also bound by RNA polymerase II [78] and it was recently reported that a fraction of extra-genic and intra-genic enhancers are actively transcribed from the H3K4me1 domain, giving rise to non-polyadenylated bidirectional transcripts called enhancer RNAs (eRNAs) [78, 79].

**Figure 2:**
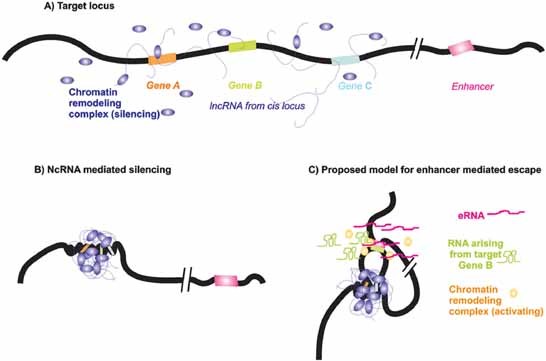
Schematic representation of lncRNA mediated silencing and proposed mechanism for escape. **A:** Hypothetical genomic locus with three genes (as labelled) regulated by a long non-coding RNA (blue) and a chromatin repressive enzyme complex (blue ovals) is shown. A distal enhancer for Gene B is represented in magenta. **B:** At the appropriate stage the lncRNA accumulates over the locus to form an lncRNA cloud. The cloud covers the genes and compacts chromatin via chromatin remodelling complexes. **C:** Proposed model for reactivation of silenced genes. A single gene is shown to escape silencing through exiting the silencing domain by looping out of the repressive compartment and physical contact with the distal enhancer. The chromatin looping may be mediated by other ncRNAs arising from the gene itself (green) in concert with eRNAs (magenta). We propose that enhancers may initiate the reactivation process through eRNAs via recruitment of chromatin activating complexes (orange spheres) or through competition for repressive complexes at the locus (not shown).

Although the function of eRNAs is as yet unclear, their expression levels are reportedly concordant with the expression levels of their target promoter transcripts [78] and their induction is reported to be a precise indication of the physical contact between enhancers and their target promoters [80]. Enhancers show developmental and activity dependent plasticity and tissue specificity [78, 81–83] indicating that they are responsive to cellular signals. Due to their physical distance from the target promoters, upon silencing of target genes via inclusion into ncRNA silencing compartments, the distal enhancers are likely to remain outside the repressive domains. Such regulatory genomic regions may be involved in mediating the escape from lncRNA mediated silencing. In particular their ability to contact promoter regions through looping of chromatin may play a role in rescuing genes out of the silent domains (Fig. 2).

An area of future research could be to investigate if genes exit the lncRNA covered silent compartment with the help of eRNAs. The fact that epigenetic modification is precisely executed at specific promoters implies that a mechanism exists within cells to facilitate recognition of specific promoters. Recent evidence indicates that double stranded short synthetic RNAs targeted at promoter regions (agRNAs) can mediate silencing as well as activation of the targeted promoters [84–87], indicating that sRNAs can recognise genomic promoters of their origin. agRNAs were shown to form a complex with AGO protein and a locally arising antisense ncRNA at the Progesterone receptor (PR) locus [88], suggesting that promoter specific RNAs may require other ncRNA mediators to execute their function. Non coding RNAs have been shown to be transcribed from active [1, 6, 15, 44, 89] as well as repressed promoters [41]. It is probable that some of these promoter specific RNAs utilise sense or antisense eRNAs as the ncRNA mediators to bring into physical proximity, silenced target genes and their enhancers via chromatin looping. These eRNA complexes may then compete with lncRNAs for the chromatin repressor complexes and facilitate reactivation of silenced genes, thus mediating exit from the repressive compartment. Alternatively, eRNAs may interact with chromatin activating complexes, and upon close proximity with target genes, induce activation through epigenetic remodelling thus mediating exit from the silencing domain (Fig. 2).

## Therapeutic applications of manipulating lncRNA mediated silencing

From the discussion above it is apparent that lncRNA mediated repression of genes is an intricate process involving chromatin remodelling enzymes and spatial reorganisation. The involvement of multiple factors suggests that the process is open to experimental manipulation at multiple levels. To be inactivated by *Xist*, genes on the X-chromosome require an abundance of LINEs in the genomic region and transcription of young LINE-1 from their vicinity [66]. In mammalian genomes, both full-length and truncated LINEs can be transcribed [90]. It is not clear what marks the imprinted genes must carry to distinguish them from non-imprinted genes within the cluster. Nevertheless the silencing and escape of genes appear to be tightly regulated and the nature of such regulation warrants investigation as it may have therapeutic applications in some disorders such as Rett Syndrome.

Rett Syndrome is an X-linked dominant neuro-developmental disorder where mutations in the *MECP2* gene cause arrest of neurodevelopment in girls [91]. Girls with Rett syndrome possess one normal and one mutant copy of the *MECP2* gene. Since *MECP2* gene undergoes X-inactivation [63], in Rett patients, the normal copy of *MECP2* gene is active only in 50% of cells while the other 50% cells express the mutant gene, which results in the phenotype. Indeed, phenotypic variations seen in Rett Syndrome are presumably dependent on the X-inactivation status of the patient [92, 93]. However, recent studies also implicate other factors [94, 95]. The *MECP2* locus on the X-chromosome is drawn inside the *Xist* silencing compartment at day 4 after differentiation [63]. Activation of the inactive non-mutant *MECP2* gene has long been proposed as a therapeutic avenue for Rett Syndrome [96]. The *Mecp2* KO male mice display striking phenotypic similarities to female patients with *MECP2* mutations [97].

Recently, Guy et al. reported a surprising reversal of the Rett phenotype seen in an experimental mouse model of Rett Syndrome by reactivation of the *Mecp2* gene in a transgenic mutant mouse. This provided the proof of principle that reactivation of the normal copy of *Mecp2* may provide therapeutic benefits in patients with loss of function *MECP2* mutations [98]. Thus a strategy of preventing the inclusion of *MECP2* in the silencing domain at early stages of differentiation or enforcing the exit of *MECP2* gene from the *XIST* silencing domain in differentiated cells may have therapeutic applications.

It will be interesting to investigate if a combined experimental approach of targeted down regulation of allele specific *MECP2* related LINE elements and allele specific over expression of *MECP2* enhancer eRNAs prevents inclusion into the silencing compartment and facilitates activation of the *MECP2* gene. It is noteworthy that the MeCP2 protein is known to repress LINE-1 transcription [99–101] and LINE-1 expression and retrotransposition is reported to be significantly higher in the adult *Mecp2* KO mouse brain as seen with genomic DNA and RNA analysis [100, 101].

In addition to Rett syndrome, manipulation of lncRNA mediated silencing may also be beneficial in preventing cancer progression, recurrence and metastasis. Gupta et al. recently reported that *HOTAIR* was over expressed up to 2,000-fold in metastatic breast tumours [102]. They demonstrated a combined role of *HOTAIR* and PRC2 complex in breast cancer invasiveness via overexpression and knock down of *HOTAIR* and PRC2 components through in vitro and in vivo studies [102]. This study indicates that *HOTAIR* and PRC2 complex specifically act through silencing of metastasis suppressor genes and alteration of the epigenetic program of breast cancer cells to promote cancer progression and metastasis. Thus selective activation of key *HOTAIR* targets, through agRNAs for example, may be beneficial in preventing invasiveness of tumours. In addition, recent studies indicate that lncRNAs, which are highly expressed in solid tumours, may be involved in cancer progression and metastasis via other mechanisms. The Metastasis Associated Lung Adenocarcinoma Transcript-1 (*MALAT-1* aka *NEAT-2*) is sequestered in nuclear speckles and is believed to alter the transcription program of cells through alternate splicing of target genes [103]. Identifying other mechanisms of ncRNA function may outline unforeseen strategies for cancer therapy.

## Future studies

To unravel the molecules involved in epigenetic regulation by lncRNAs, it is important to first identify genes regulated by lncRNAs. Global investigation of chromatin-RNA interactions at different developmental stages is essential. Combined sequencing of RNA and DNA molecules in close proximity on a genomic scale will aid the discovery of chromatin-RNA associations at different development stages. Although chromatin associated RNAs (CARs) were recently sequenced on a genome wide scale, the exact region of their association with chromatin has not been investigated [104]. The identification of the region of chromatin interaction is essential for the discovery of targets since ncRNAs do not always associate with genomic regions of sequence homology. Gene expression analysis using high throughput quantitative techniques such as CAGE [105] conducted in parallel will identify ncRNA chromatin interactions resulting in activation or repression of genes. Thus new candidate ncRNAs likely to create silencing domains or participate in the activation of genes can be identified and individually investigated.

It is also necessary to identify the distal enhancers of genes regulated by ncRNAs. Although some recent studies have identified enhancers in neurons [78] and cardiomyocytes [83], since enhancers are tissue and development stage specific, there is a need to perform ChIP sequencing using antibodies against specific markers such as P300, H3K4me1, H3K27ac and histone variant H2A.Z in various tissues to identify tissue specific enhancers on a global scale. In addition, technologies such as HiC and ChIA-PET, which can identify genome wide chromatin-chromatin associations, have the capability to identify distal enhancers in physical contact with promoters [106, 107]. The HiC technique is based on proximity ligation and provides unbiased genome-wide maps of chromatin-chromatin association [106]. The CHIA-PET technique, based on immunoprecipitation, proximity ligation and paired end tag sequencing [107] will be especially useful in the identification of enhancers, if performed with antibodies against P300, H3K4me1 and H3K27ac.

It is also important to catalogue lncRNAs and permit their search on public genome browsers. Although some lncRNAs are viewable on public genome browsers and lncRNAdb, thousands of human lncRNAs and expressed retrotransposons identified in FANTOM3 [1, 90], lincRNAs identified in other projects using the k4-36 signature [108] and later with the RIP-seq assay [26] are not clearly annotated on the public browsers.

Thus we remain unaware of the regulatory lncRNAs expressed from the genomic vicinity of our genes of interest. Given the role of lncRNAs in gene regulation, the availability of dedicated tracks of full-length lncRNAs derived from FANTOM3, FANTOM5, ENCODE [1, 109, 110] and other similar projects would be of immense benefit to biologists seeking answers to gene regulation. Since most regulatory lncRNAs are likely to be nuclear, as major transcriptome sequencing projects such as ENCODE and FANTOM5 progress, additional tracks in public browsers based on the distribution of these lncRNAs such as nuclear, cytoplasmic, nucleoplasm or chromatin associated etc will speed up experimental validation of lncRNA related hypotheses.

## Conclusions and perspectives

The common themes in the mechanism of silencing mediated by lncRNAs, such as Xist, HOTAIR, Kcnq1ot1 and Airn, have provided a sound template for the investigation of other similar molecules in cells, whose function remains unknown. Although in this review we have focused on the silencing aspect of lncRNA function, evidence is now emerging of lncRNAs participating in gene activation during chromatin looping [111]. Furthermore, novel gene specific mechanisms of silencing are also being uncovered [50].

Thus, it is clear that much remains to be learned in the field of lncRNA function. In addition, another layer of regulation appears to exist at the cellular level, which dictates the transcriptional program, by specifying lncRNA targets. This regulatory layer appears to be tissue and development stage specific and concerted efforts are needed to decipher this next level of control. Just as the lncRNAs have similarities in their modes of action, it is likely that the additional layer of regulatory control over lncRNA mediated silencing may also have common mechanisms. Whether distal enhancers, ncRNAs or other protein complexes exercise this control remains to be investigated. In the near future we may unravel universal techniques to reverse or enforce epigenetic silencing of specific targets mediated by lncRNAs, providing novel therapeutic avenues for some disorders.

## Abbreviations:

agRNA: antigene RNAs
lincRNAs: long intergenic non-coding RNAs
lncRNAs: long non-coding RNAs
LINEs: long interspersed nuclear elements
ncRNAs: non-coding RNAs
siRNA: short interfering RNA
